# *TRAF1/C5* locus is associated with response to anti-TNF in rheumatoid arthritis

**DOI:** 10.1186/1479-5876-10-S3-P31

**Published:** 2012-11-28

**Authors:** Helena Canhão, Ana Rodrigues, Maria Jose Santos, Diana Carmona-Fernandes, José Costa, Helena Santos, Jaime Branco, Robert Plenge, Daniel Solomon, Jacome Armas, José António Silva, João Eurico Fonseca, Elizabeth Karlson

**Affiliations:** 1 Rheumatology Research Unit, Instituto Medicina Molecular, Lisbon, Portugal; 2 Rheumatology Dept., Unidade Saúde Alto Minho, PonteLima, Portugal; 3 Instituto Português Reumatologia, Lisbon, Portugal; 4 CEDOC, Lisbon, Portugal; 5 Division of Rheumatology, Brigham and Women's Hospital, Boston, USA; 6 SEEBMO, Azores, Portugal; 7 Rheumatology Dept., Centro Hospitalar Universidade Coimbra, Coimbra, Portugal

## Introduction

Some of the rheumatoid arthritis (RA) risk allele variants were related to tumor necrosis factor (TNF) signaling pathways which raised the hypothesis that they might influence the response to anti-TNF drugs.

The primary aim of our work was to investigate potential association between the *HLA-DRB1* and RA risk alleles specifically selected for their relevance on RA biologic pathways with the response to anti-TNF treatment in a Southern European population using a nationwide register.

## Methods

We evaluated 383 RA patients for associations between anti-TNF treatment response assessed by an absolute change in DAS28 at six months as the primary outcome, and seven single nucleotide polymorphisms (SNP). We also studied the same association taking the proportion of EULAR good responders and non responders at six months as the outcome. Univariate and multivariate linear and logistic regression analyses were performed, adjusting for clinical variables that influenced treatment response.

## Results

The minor allele (G), which is the risk allele for RA susceptibility, rs3761847 SNP in the *TRAF1/C5* region was associated with a poor anti-TNF treatment response either in linear (coefficient -0.24; 95% confidence interval (CI) -0.43, -0.06; p-value 0.009) and in logistic univariate (odds ratio (OR) 0.61; CI 0.41, 0.92; p-value 0.018) and multivariate regression analyses.

Associations between *HLA-DRB1* or the other allele variants with the response to anti-TNF treatment were not observed.

## Conclusion

The rs3761847 *TRAF1/C5* risk RA locus influenced the anti-TNF treatment response in the Southern European population assessed in this study. Additional studies in other populations are necessary to confirm the relevance of this finding.

